# Early and late gut microbiota signatures of stroke in high salt-fed stroke-prone spontaneously hypertensive rats

**DOI:** 10.1038/s41598-024-69961-9

**Published:** 2024-08-23

**Authors:** Silvia Bencivenni, Sara Roggiani, Augusta Zannoni, Gabriele Conti, Marco Fabbrini, Maria Cotugno, Rosita Stanzione, Donatella Pietrangelo, Margherita Litterio, Maurizio Forte, Carla Letizia Busceti, Francesco Fornai, Massimo Volpe, Silvia Turroni, Patrizia Brigidi, Monica Forni, Speranza Rubattu, Federica D’Amico

**Affiliations:** 1 Unit of Microbiome Science and Biotechnology, Department of Pharmacy and Biotechnology, University of Bologna, Via Belmeloro 6, 40126 Bologna, Italy; 2 Department of Veterinary Medical Sciences, University of Bologna, Ozzano dell’Emilia, Bologna, Italy; 3 Department of Medical and Surgical Sciences, University of Bologna, Bologna, Italy; 4 Health Sciences and Technologies-Interdepartmental Center for Industrial Research (CIRI-SDV), Alma Mater Studiorum-University of Bologna, 40126 Bologna, Italy; 5 IRCCS Neuromed, Pozzilli, Isernia Italy; 6 Department of Clinical and Molecular Medicine, School of Medicine and Psychology, Sapienza University of Rome, Rome, Italy; 7 Department of Translational Research and New Technologies in Medicine and Surgery, University of Pisa, Pisa, Italy; 8 IRCCS San Raffaele, Rome, Italy

**Keywords:** Stroke, Gut microbiota, High-salt diet, SHRSP, Gut barrier, Microbial ecology, Microbiome, Hypertension, Stroke

## Abstract

The high salt-fed stroke-prone spontaneously hypertensive rat (SHRSP) is a suitable tool to study the mechanisms underlying stroke pathogenesis. Salt intake modifies the gut microbiota (GM) in rats and humans and alterations of the GM have previously been associated with increased stroke occurrence. We aimed to characterize the GM profile in SHRSPs fed a high-salt stroke-permissive diet (Japanese diet, JD), compared to the closely related stroke-resistant control (SHRSR), to identify possible changes associated with stroke occurrence. SHRSPs and SHRSRs were fed a regular diet or JD for 4 weeks (short-term, ST) or a maximum of 10 weeks (long-term, LT). Stroke occurred in SHRSPs on JD-LT, preceded by proteinuria and diarrhoea. The GM of JD-fed SHRSPs underwent early and late compositional changes compared to SHRSRs. An overrepresentation of *Streptococcaceae* and an underrepresentation of *Lachnospiraceae* were observed in SHRSPs JD-ST, while in SHRSPs JD-LT short-chain fatty acid producers, e.g. *Lachnobacterium* and *Faecalibacterium*, decreased and pathobionts such as *Coriobacteriaceae* and *Desulfovibrio* increased. Occludin gene expression behaved differently in SHRSPs and SHRSRs. Calprotectin levels were unchanged. In conclusion, the altered GM in JD-fed SHRSPs may be detrimental to gut homeostasis and contribute to stroke occurrence.

## Introduction

The gut microbiota (GM) refers to the large and diverse community of microorganisms, mainly bacteria, that reside in the gastrointestinal tract, and whose relevance to host physiology is widely recognized^1^. Alterations of this community (i.e*.* dysbiosis) have been shown to contribute to the development and progression of several intestinal and extra-intestinal diseases, including cardiovascular diseases (CVDs), in both animal models^1^ and humans^1^. The diet-microbiota-host axis can promote atherosclerosis^2,3^ and is involved in the development of hypertension, metabolic syndrome, stroke and its outcomes^4–6^. Transplantation of caecal contents from spontaneously hypertensive rats (SHR) induced hypertension in recipient normotensive rats^4^. Consistently, GM transplantation from the stroke-prone spontaneously hypertensive rat (SHRSP) increased blood pressure (BP) in the recipient normotensive Wistar Kyoto (WKY) strain^4^. Furthermore, a previous study showed that the SHRSP GM was altered in structure and function with aging compared to the genetically normotensive control WKY strain^7^. Interestingly, intermittent fasting corrected gut dysbiosis and lowered BP levels in SHRSP, with bile acids acting as potential mediators in the microbe-host interactome involved in BP regulation^8^.

The SHRSP animal model represents a suitable experimental tool for studying the human disease. SHRSPs develop spontaneous hypertension and target organ damage, mainly renal and cerebrovascular damage, with aging^9^. Feeding SHRSPs with a high-salt/low-potassium diet (Japanese style diet, JD) accelerates the development of hypertensive target organ damage (vasculitis type) compared to the closely related control strain, the stroke-resistant SHR (SHRSR), despite similar BP levels^10^. Stroke occurrence is regularly anticipated in SHRSPs by the appearance of proteinuria, weight loss and often diarrhoea^11^. The latter may support the involvement of GM dysbiosis, inflammation and increased permeability in the higher stroke predisposition of this strain. Consistent with this, a recent work reported that the GM contributes to blood–brain barrier (BBB) disruption in SHRSPs^12^. In fact, BBB integrity was improved in SHRSPs fostered on WKY dams compared to SHRSPs fostered on their own dams, suggesting that environmental factors such as the GM of the foster dam may regulate it^12^. Of note, high salt intake, which favours stroke in SHRSP, caused changes in the GM and induced hypertension in normotensive rats^13^. High salt intake also induced significant changes in the human GM^14^. However, evidence on the possible pathogenic contribution of dysbiosis to the higher stroke predisposition of high salt fed SHRSPs is still lacking. In the present study, we aimed to explore this issue and to identify the alterations of the GM induced by the high-salt stroke permissive diet (JD) in SHRSPs compared to SHRSRs, both before stroke appearance and at the time of stroke occurrence. We also assessed inflammatory status by measuring the serum levels of calprotectin and the gut barrier integrity by assessing gene expression of the tight-junction proteins zonulin (ZO-1*)* and occludin (Ocln).

## Results

### Phenotypic parameters of SHRSRs and SHRSPs after short-term (4 weeks) JD feeding

**Figure 1:**
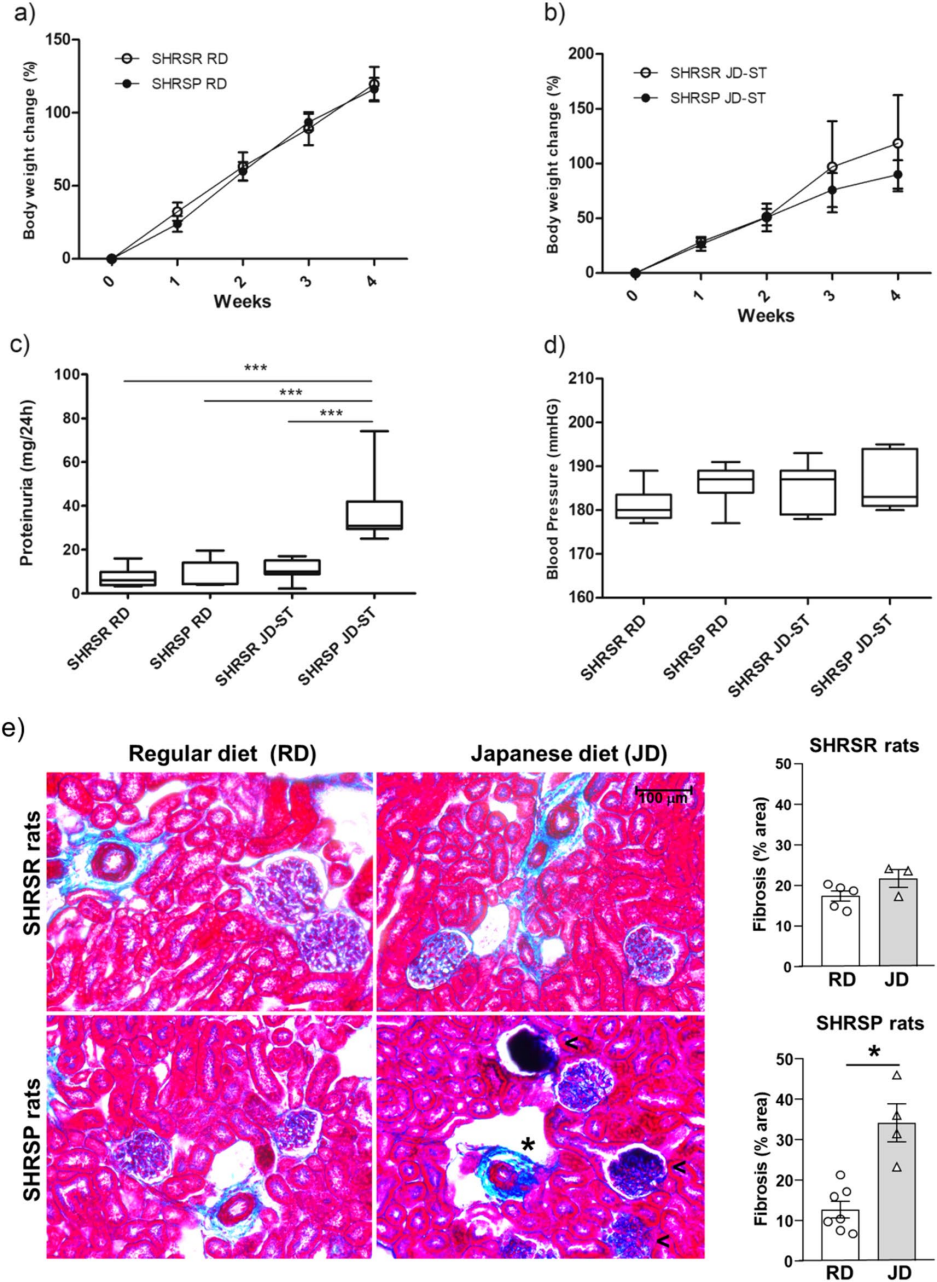
Phenotypic parameters of SHRSRs and SHRSPs after short-term (ST, 4 weeks) regular diet (RD) or Japanese diet (JD). Body weight change (%) during 4 weeks of RD (**a**) or JD (**b**) feeding in the two rat strains. (**c**) Proteinuria levels after 4 weeks of RD or JD feeding in the two rat strains. (**d**) Systolic blood pressure levels at the same experimental times. Two-way ANOVA followed by Tukey post-hoc comparison. In panels (**a**) and (**b**), only different rat strains at the same time point were statistically compared. SHRSR RD, n = 8 in panel a, n = 9 in panel (**c**) and (**d**); SHRSP RD, n = 7; SHRSR JD-ST, n = 7; SHRSP JD-ST, n = 7. (**e**) Azan Masson’s trichrome staining showed a significant increase in perivascular (*) and glomerular ( <) fibrosis in SHRSPs, but not SHRSRs, fed JD for 4 weeks compared with the corresponding controls fed RD. Bar graphs show quantification of fibrosis as percentage of blue. Data are mean + /– standard error of the mean (SEM). Mann–Whitney two-tailed test. *p < 0.05, **p < 0.01, ***p < 0.001.

Body weight (BW) increased significantly in a comparable manner in SHRSRs and SHRSPs (p < 0.001, two-way ANOVA) over 4 weeks (short term, ST) of JD (JD-ST) or control diet (regular diet, RD) feeding (Fig. 1a,b), but it was generally higher in SHRSR animals compared to SHRSP animals upon the same diet. However, there was not a significant difference in BW between different rat strain fed with the same diet for the same number of weeks. Initial weights of animals are available in Supplementary Table S1. Proteinuria levels were significantly increased in SHRSPs JD-ST compared to RD-fed SHRSPs, RD-fed SHRSRs and SHRSRs JD-ST (p < 0.001, two-way ANOVA) (Fig. 1c). Systolic blood pressure (BP) was not significantly different among the four groups (Fig. 1d). No signs of stroke occurred during the 4-week period in either JD or RD-fed rats. Renal histology revealed the presence of damage in SHRSPs as previously reported^15–17^ (Fig. 1e).

### Phenotypic parameters of SHRSRs and SHRSPs on long-term JD feeding

**Figure 2:**
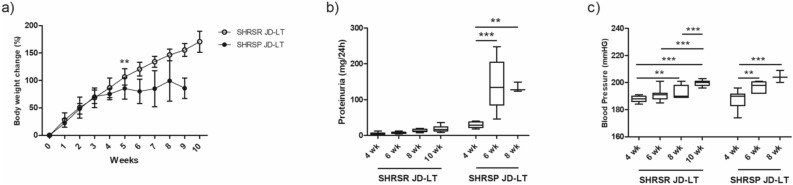
Phenotypic parameters of SHRSRs and SHRSPs under long-term Japanese diet (JD-LT) feeding. (**a**) Body weight change (%) during long-term JD feeding in the two rat strains. Two-way ANOVA followed by Tukey post-hoc comparison calculated only untill the first rat died of stroke (after 6 weeks). Only different rat strains at the same time point were statistically compared. SHRSR JD-LT, n = 11; SHRSP JD-LT, n = 13 up to week 5; SHRSP JD-LT, n = 10 at week 6; SHRSP JD-LT, n = 5 at week 7; SHRSP JD-LT, n = 3 at week 8; SHRSP JD-LT, n = 2 at week 9. (**b**) Proteinuria and (**c**) blood pressure levels after long-term JD feeding in the two rat strains. One-way ANOVA followed by Tukey post-hoc comparison. SHRSR JD-LT, n = 11 at all timepoints; SHRSP JD-LT, n = 13 at week 4; SHRSP JD-LT, n = 9 at week 6; SHRSP JD-LT, n = 3 at week 8. **p < 0.01, ***p < 0.001.

The increase in BW was different between the two rat groups fed long-term (LT) JD, being significantly higher in SHRSRs JD-LT than in SHRSPs JD-LT from week 5 of JD (p < 0.001, two-way ANOVA followed by Tukey post-hoc comparison) (Fig. 2a). Proteinuria levels significantly increased in SHRSPs JD-LT after 6 weeks of JD (p < 0.001, one-way ANOVA followed by Tukey post-hoc comparison). In contrast, proteinuria levels did not change in SHRSRs JD-LT (Fig. 2b). Systolic BP increased significantly over time in both SHRSRs JD-LT and SHRSPs JD-LT (Fig. 2c).

**Figure 3:**
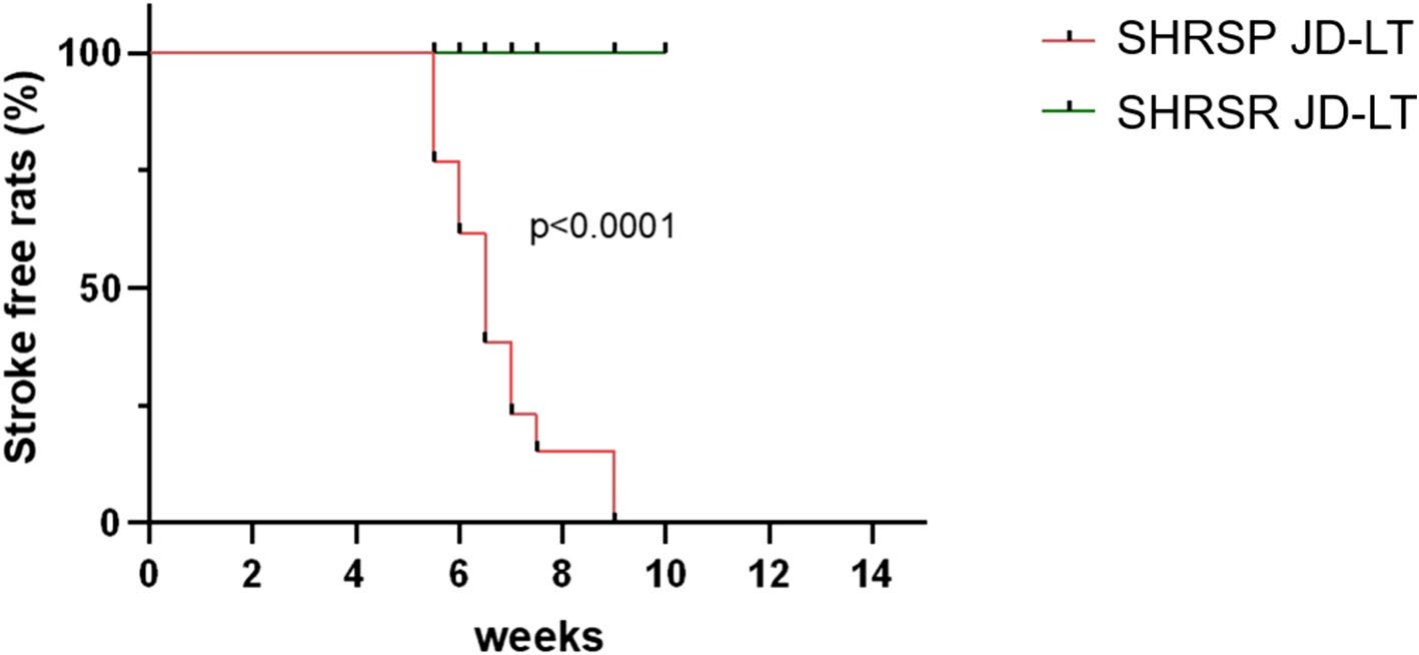
Stroke occurrence in SHRSRs and SHRSPs upon long-term JD feeding. Percentage of stroke events in the two rat strains upon JD-LT treatment. The comparison between the two strains was significantly different (Log-rank and Mann–Whitney test, p < 0.0001). For the number of animals included in this analysis, see Fig. 2.

Stroke started to occur in SHRSPs JD-LT after 6 weeks of JD feeding (25% incidence), and its incidence increased to 100% at week 9 of JD feeding (Fig. 3). Stroke was manifested by signs of limb paresis/hemiparesis, epileptic attacks, and sudden death, and it was always preceded by diarrhea and proteinuria. Stroke never occurred in SHRSRs JD-LT^10,11^.

### Gut microbiota profile in JD-fed SHRSRs and SHRSPs

The large intestinal microbiota was profiled in SHRSRs and SHRSPs after 4 weeks of RD or JD feeding (JD-ST) and after long-term JD feeding (JD-LT). No differences in alpha diversity were found between the study groups (Supplementary Fig. S1). Similarly, no segregation between groups was found in the PCoA plots of unweighted and weighted UniFrac distances (Supplementary Fig. S1).

**Figure 4:**
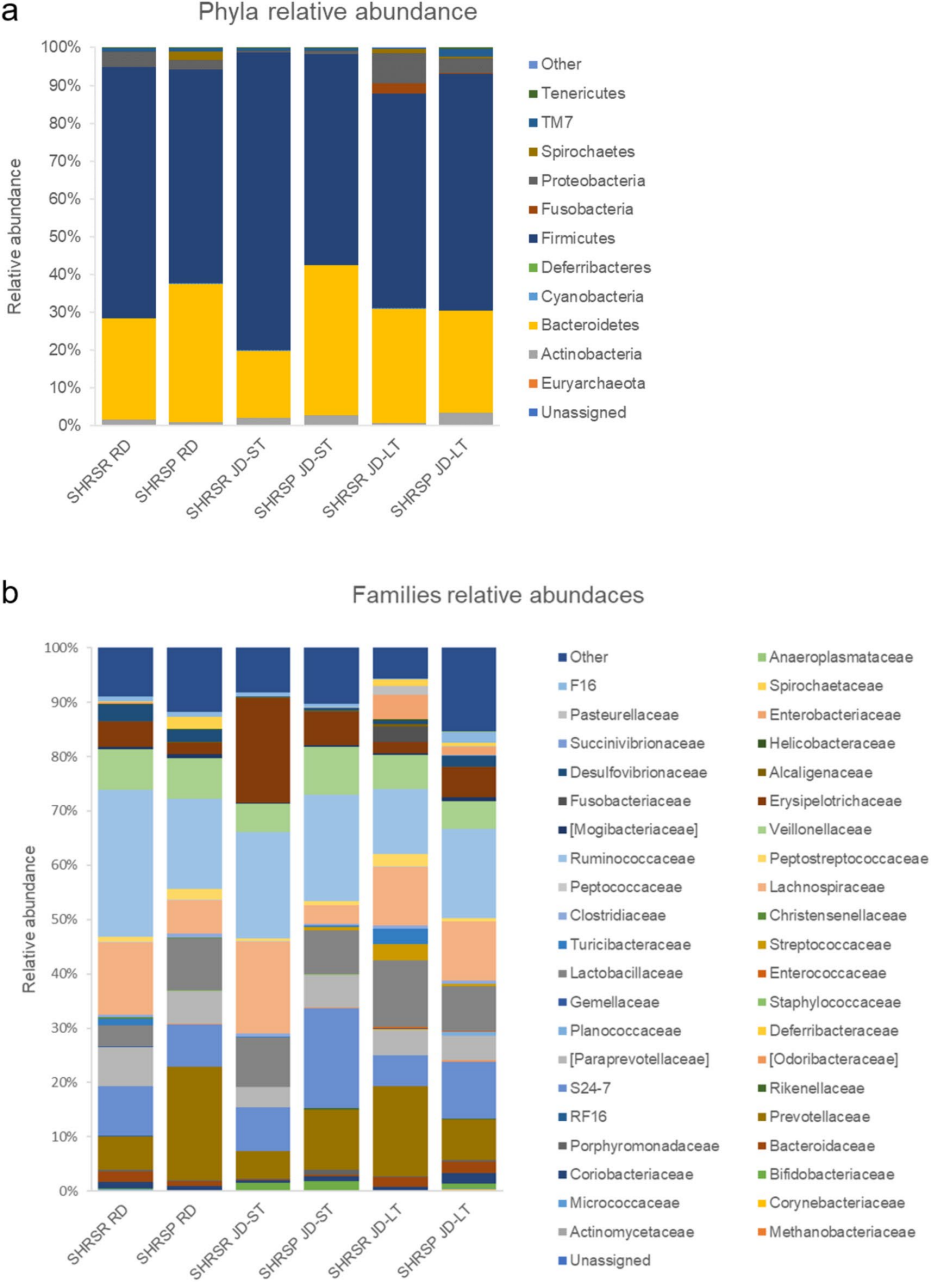
Phylum and family-level gut microbiota composition of SHRSRs and SHRSPs after 4 weeks of regular diet (RD) or Japanese diet (JD-ST), and after long-term JD feeding (JD-LT). Bar plots showing the relative abundance of major phyla (**a**) and families (**b**) in the large intestine of the two rat strains after 4 weeks of RD or JD feeding (JD-ST) and after long-term JD feeding (JD-LT). Only taxa with a relative abundance > 0.1% in at least 3 samples are shown. SHRSR RD, n = 9; SHRSP RD, n = 7; SHRSR JD-ST, n = 8; SHRSP JD-ST, n = 7; SHRSR JD-LT, n = 11; SHRSP JD-LT, n = 13.

From the taxonomic standpoint, the GM of all groups was largely dominated by the phyla Firmicutes (mean ± SEM in the whole cohort, 62.8% ± 2.1) and Bacteroidetes (29.1% ± 2.2), with *Ruminococcaceae* (18.1% ± 1.3), *Prevotellaceae* (10.9% ± 1.5), *Lachnospiraceae* (10.6% ± 1.1), *S24-7* (9.6% ± 1.0) and *Lactobacillaceae* (8.6% ± 1.3) being the most relatively abundant families (Fig. 4).

**Figure 5:**
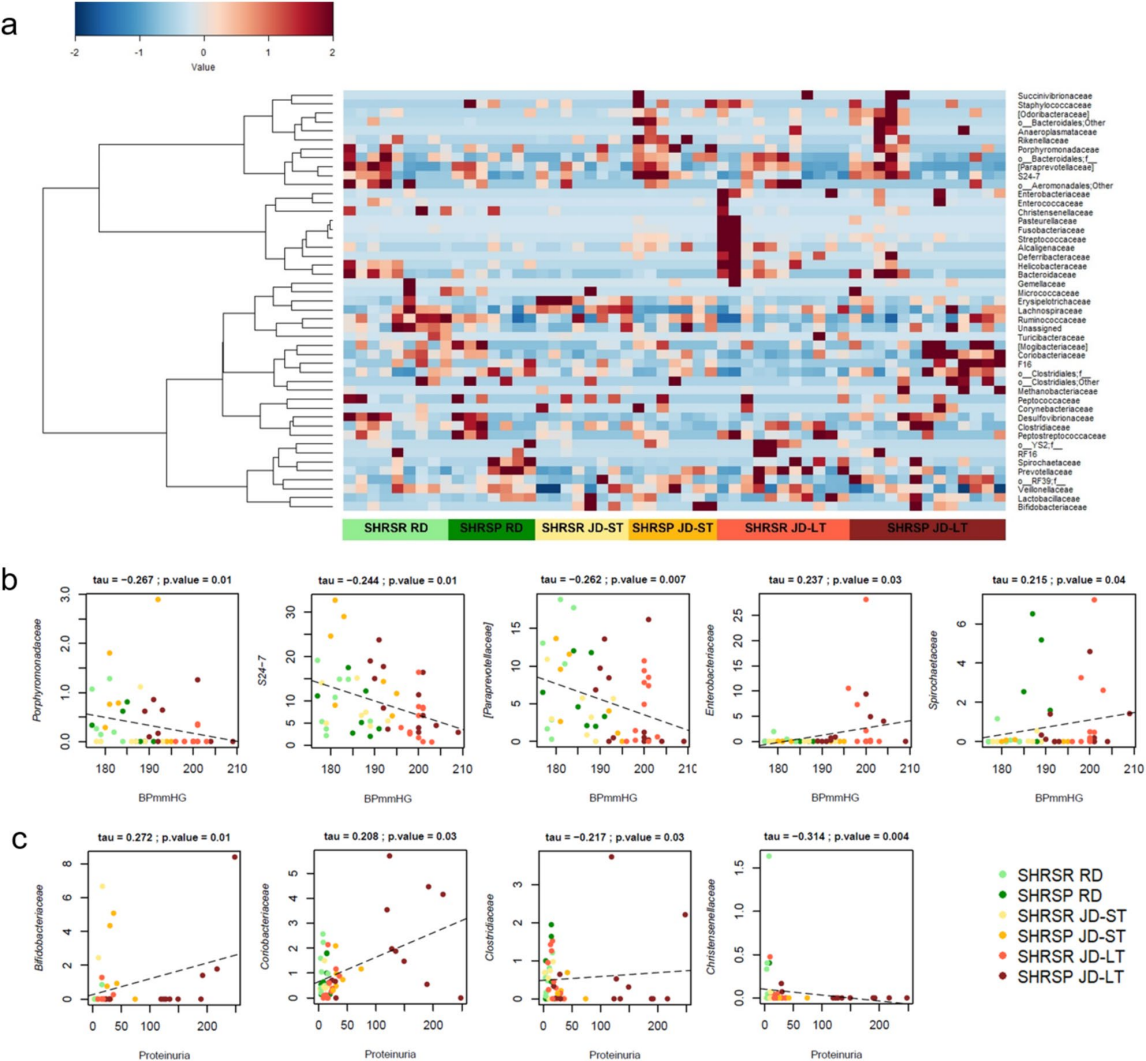
Family-level gut microbiota signatures of SHRSRs and SHRSPs after 4 weeks of regular diet (RD) or Japanese diet (JD-ST), and after long-term JD feeding (JD-LT), and correlations with blood pressure and proteinuria levels. (**a**) Heatmap showing Ward-linkage clustering based on Pearson's correlation coefficients of the relative abundance of gut microbiota families of the two rat strains after 4 weeks of RD or JD feeding (JD-ST) and after long-term JD feeding (JD-LT). Only taxa with a relative abundance > 0.1% in more than 2 samples are shown. See Supplementary Fig. S2 for further details. Scatter plots of correlation between relative abundance of families and (**b**) blood pressure (BPmmHG) and (**c**) proteinuria levels in SHRSRs and SHRSPs after RD or JD-ST/JD-LT. Only statistically significant correlations (p ≤ 0.05) based on Kendall rank correlation test with absolute tau ≥ 0.2 are shown.

Notably, JD-fed SHRSRs and SHRSPs differed in the proportions of several taxa in both ST and LT (Fig. 5a and Supplementary Figs. S2 and S3). The SHRSP JD-ST group was characterized by a significantly higher relative abundance of the phyla Bacteroidetes and Proteobacteria, and a lower relative abundance of Firmicutes compared to SHRSRs JD-ST (p < 0.05, Mann–Whitney *U* test), suggesting an early strain-dependent high-level disruption of the GM. SHRSPs JD-ST also showed a significant overrepresentation of the family *Streptococcaceae* (and its genus *Streptococcus*) and an underrepresentation of *Lachnospiraceae* (especially *Blautia*) compared to the SHRSR counterpart (p < 0.05). It should be noted that *Blautia* proportions were significantly higher in both SHRSRs JD-ST and JD-LT compared to RD-fed SHRSRs (p < 0.05), suggesting a dietary and strain-related effect. The main discriminating taxa between the two rat strains JD-LT were the phylum Actinobacteria and the families *Coriobacteriaceae*, *Erysipelotrichaceae* (and its genus *Allobaculum*) and *Desulfovibrionaceae* (and *Desulfovibrio*), which were enriched in SHRSPs. In contrast, the families *Peptostreptococcaceae*, *Alcaligenaceae* (and *Sutterella*), *Helicobacteraceae* and *Turicibacteraceae* (and *Turicibacter*), and the genera *[Prevotella]*, *Candidatus Arthromitus*, *Lachnobacterium* and *Faecalibacterium* were significantly enriched in SHRSRs (p < 0.05). Again, diet and strain-dependent patterns were found for some taxa, namely Actinobacteria, *Erysipelotrichaceae* and *Sutterella*, whose increases were also significant compared to the RD-fed counterparts (p < 0.05). See Fig. 5a and Supplementary Figs. S2 and S3 for all significant differences between the study groups, including those between the two RD-fed rat strains. When looking for correlations between relative taxon abundance and host metadata, we found that BP was negatively correlated with the families *Porphyromonadaceae*, *S24-7* and *[Paraprevotellaceae]* (tau ≤ − 0.244, p ≤ 0.01, Kendall rank correlation test), while a positive correlation was found with *Enterobacteriaceae* and *Spirochaetaceae* (tau ≥ 0.215, p ≤ 0.04) (Fig. 5b). Positive associations were also observed for *Bifidobacteriaceae* and *Coriobacteriaceae* with proteinuria levels (tau ≥ 0.208, p ≤ 0.03), while *Clostridiaceae* and *Christensenellaceae* were negatively correlated (tau ≤ − 0.217, p ≤ 0.03) (Fig. 5c).

### Serum calprotectin

**Figure 6:**
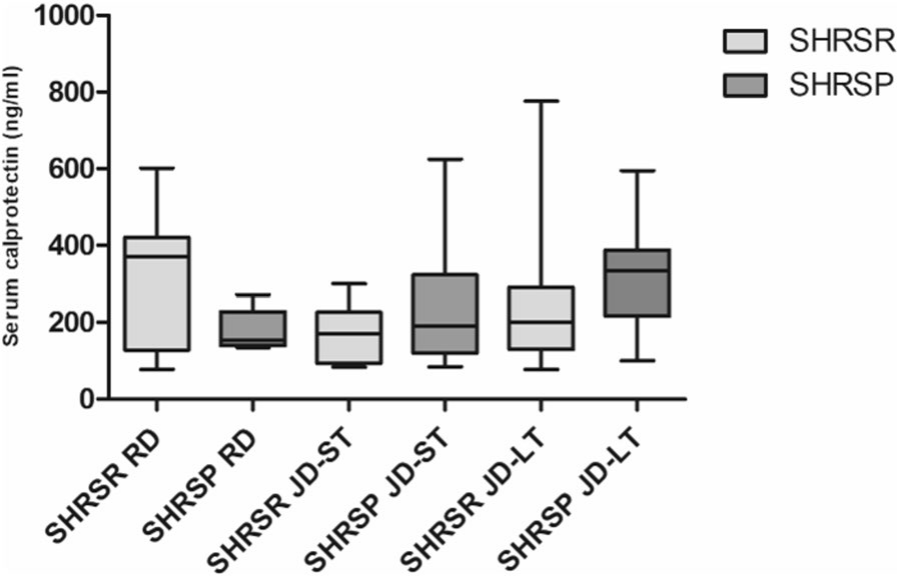
Calprotectin concentration in the serum of SHRSRs and SHRSPs after 4 weeks of regular diet (RD) or Japanese diet (JD-ST), and after long-term JD feeding (JD-LT). Values in ng/ml (mean ± SD) are shown for the two rat strains after 4 weeks of RD or JD feeding (JD-ST) and after long-term JD feeding (JD-LT). SHRSR RD, n = 7; SHRSP RD, n = 5; SHRSR JD-ST, n = 7; SHRSP JD-ST, n = 6; SHRSR JD-LT, n = 10; SHRSP JD-LT, n = 13.

Serum calprotectin levels ranged from 77.9 to 775.9 ng/ml (Fig. 6). No statistically significant difference was observed between the study groups. Three out of the 50 samples analyzed were excluded because they were below the detection limit.

### ZO-1 and Ocln relative gene expression in the small intestinal mucosa

RT-qPCR of *ZO-1* yielded measurable cycle threshold (Ct) levels in 88 out of 110 wells, while *Ocln* expression analysis yielded 104 out of 110 measurable Ct levels (samples in duplicate).

**Figure 7:**
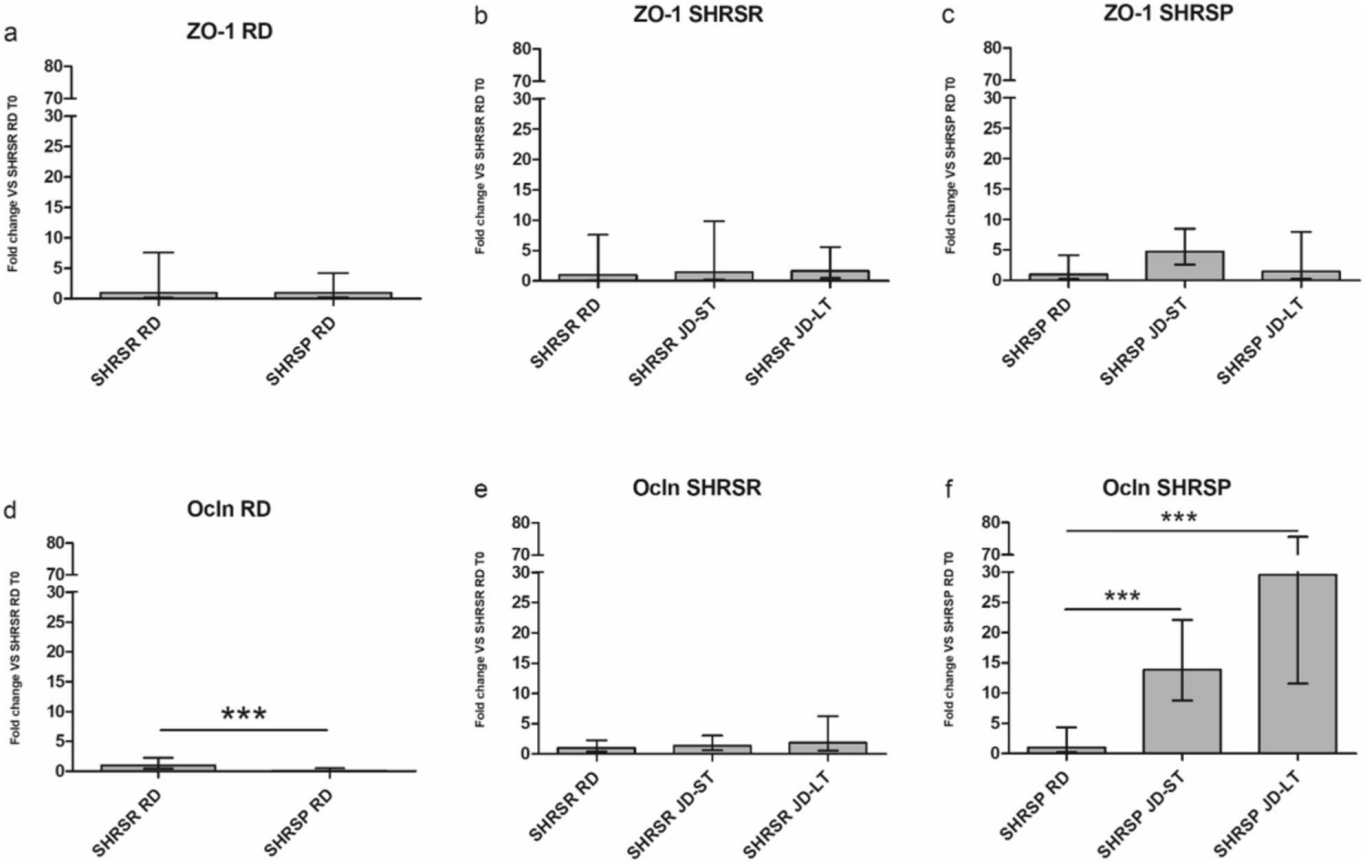
Relative gene expression of ZO-1 and Ocln in the small intestinal mucosa of SHRSRs and SHRSPs after 4 weeks of regular diet (RD) or Japanese diet (JD-ST), and after long-term JD feeding (JD-LT). Relative expression was calculated as fold change with respect to SHRSR RD or SHRSP RD groups. The error bar represents the range of relative expression. *p < 0.05, **p < 0.01, ***p < 0.001, (**a**–**d**) *t*-test; (**e**–**f**) two-way ANOVA, post-hoc standard with Tukey correction. SHRSR RD, n = 9; SHRSP RD, n = 7; SHRSR JD-ST, n = 8; SHRSP JD-ST, n = 7; SHRSR JD-LT, n = 11; SHRSP JD-LT, n = 13.

No changes in ZO-1 mRNA expression levels were detected among the two strains and the two dietary regimens (Fig. 7a–c, *t*-test). In contrast, Ocln mRNA levels were significantly lower in RD-fed SHRSPs compared to RD-fed SHRSRs (p < 0.001) (Fig. 7d) and significantly increased in SHRSPs JD-ST and JD-LT compared to RD-fed counterparts (p < 0.001) (Fig. 7f, two-way ANOVA). In contrast, Ocln levels were not higher in JD-fed SHRSRs compared to RD-fed SHRSRs (Fig. 7e). Two-way ANOVA confirmed an interaction between diet and strain both after 4 weeks of diet (RD vs. JD-ST) and at the end of the experiment (RD vs. JD-LT, p < 0.001).

## Discussion

The present study provides the first GM profiling in SHRSPs fed with a high-salt stroke-permissive dietary regimen compared to the closely related stroke-resistant control (SHRSR). The JD-fed SHRSP represents a suitable experimental tool to investigate molecular mechanisms underlying stroke predisposition with a potential relevance for the human disease. Furthermore, based on the available literature, high salt intake elicits alterations in the GM of rats^13^, and this happens in a strain dependent manner for certain taxa.

In this study we found that JD-fed SHRSPs exhibited both early and late alterations in the GM composition compared to SHRSRs. Specifically, after short-term JD feeding, a critical time window that triggers the following stroke events, SHRSPs showed a high-taxonomic level rearrangement involving the major phyla Firmicutes and Bacteroidetes, with an overrepresentation of *Streptococcaceae* (and *Streptococcus*) and an underrepresentation of *Lachnospiraceae* (especially *Blautia*). Of note, *Streptococcus* has been previously found to be overabundant in hypertensive subjects and it has been proposed as a cause of increased vasoconstriction through increased serotonin production and consequent excessive sympathetic activation^18^. Some *Streptococcus* spp. have also been found to be increased in the GM following stroke^19^, strengthening their association with vascular events and outcomes. The variation observed inSHRSPs with regard to *Lachnospiraceae* members is also consistent with the available literature in hypertensive subjects^20^. Furthermore, *Lachnospiraceae* members are undoubtedly associated with healthy dietary patterns since their main metabolites, short-chain fatty acids, have been shown to regulate BP, to improve microcirculation, and to exert anti-inflammatory effects^21,22^.

Notably, after long-term JD feeding, the above-mentioned changes were no longer observed, suggesting a possible predominant effect of the diet. However, SHRSPs and SHRSRs still exhibited distinct microbiota compositional features, indicating the persistence of strain-specific patterns that may correlate with stroke occurrence upon prolonged JD feeding. These features included an increase of Actinobacteria (and of its family *Coriobacteriaceae*), *Erysipelotrichaceae* (and of its genus *Allobaculum*) and *Desulfovibrionaceae* (and *Desulfovibrio*) in SHRSPs. In contrast, *Peptostreptococcaceae*, *Alcaligenaceae* (and *Sutterella*), *Helicobacteraceae*, *Turicibacteraceae* (and *Turicibacter*), *[Prevotella]*, *Candidatus Arthromitus*, *Lachnobacterium* and *Faecalibacterium* increased in SHRSRs. Again, few variations were expected, such as the SHRSP-related decrease in the short-chain fatty acid producers *Turicibacter*, *Lachnobacterium* and *Faecalibacterium*, and the SHRSP-related increase in the pathobionts *Coriobacteriaceae* and *Desulfovibrio*. In particular, *Coriobacteriaceae* members were found to be overrepresented in patients with symptomatic atherosclerosis^23^. Notably, the relative abundance of *Coriobacteriaceae* was positively correlated with proteinuria levels in our cohort^24,25^. Moreover, *Desulfovibrio* was found in hypertensive subjects and in patients with large-artery atherosclerotic ischemic stroke and transient ischemic attack^19,26^. Interestingly, the *Desulfovibrio* pathogenicity is likely related to excessive production of H_2_S, which could be deleterious in ischemic stroke by impairing mitochondrial function and inducing neurotoxicity^27^. The latter effects may be consistent with the known mitochondrial dysfunction observed in SHRSPs^28^ and may support the development of the small vessel cerebrovascular disease of the strain. On the other hand, the increase of *Allobaculum* in SHRSPs contrasts with the available evidence, which shows an association between this mucin-degrading taxon and lower BP in SHRs^29^, as well as improved hypertension-induced endothelial dysfunction^30^.

Altogether, our study highlighted a different behavior of certain taxa in the SHRSP vs. SHRSR strains upon the stroke-permissive diet, and some of these variations could be consistent with a contributory pathogenetic role. Based on our current findings, future studies may assess the impact of either microbiota depletion through antibiotic treatment or fecal transplantation on stroke occurrence in SHRSPs.

Regarding intestinal permeability, the available evidence in stroke pathogenesis is limited and contradictory. Some studies in mice have shown that the small intestinal permeability is increased because of decreased expression of tight-junction proteins (ZO-1 and Ocln)^31^, while others have shown that stroke does not alter intestinal shape and function^32^. Low levels of ZO-1 and Ocln were found in SHRSRs with established hypertension compared with normotensive controls (Wistar Kyoto rats)^33^. In our study, ZO-1 expression did not change between the two rat strains on the two diets. In contrast, Ocln expression was significantly lower in SHRSPs compared to SHRSRs upon RD and increased in a time-dependent manner only in JD-fed SHRSPs, suggesting a strain-dependent interaction with the diet. The increase of Ocln could be explained as a compensatory mechanism to the diet-induced intestinal derangements, likely due to increased proteasome-mediated degradation^34^, and it may support the involvement of reduced gut permeability in stroke pathogenesis.

Inflammation is recognized as an important element in the pathogenesis of ischemic stroke. In previous studies, circulating calprotectin levels were found to be elevated in acute ischemic stroke^35^ and after ischemic stroke onset^36^, possibly predicting 3-month mortality^37^. Although SHRSPs carry a vasculitis-like cerebrovascular damage, no strain/diet-related changes in serum calprotectin levels were detected in our study.

The main limitations of the present study include: (i) the small number of animals used; (ii) the lack of more frequent longitudinal sampling to reconstruct the temporal dynamics of changes in the microbiota; (iii) the associative nature of the experimental design, which did not involve mechanistic investigation; and (iv) the use of 16S rRNA amplicon sequencing instead of shotgun metagenomics, which did not allow high-resolution taxonomic and functional profiling.

In conclusion, our study showed that the GM of JD-fed SHRSPs undergoes both early and late compositional changes that differ from their SHRSR counterparts, some of which may be detrimental to gut homeostasis and may contribute to the occurrence of stroke. These hypothesis-generating findings need to be confirmed in larger sample sets, possibly collected over time, and analysed using different approaches, including omics and functional analysis, to elucidate the role of the identified microbial signatures in stroke pathogenesis. A deeper understanding of the underlying molecular mechanisms may pave the way for future GM-targeted preventive and therapeutic strategies against hypertension and its consequences, including stroke.

## Materials and methods

### Experimental design and sampling

**Figure 8:**
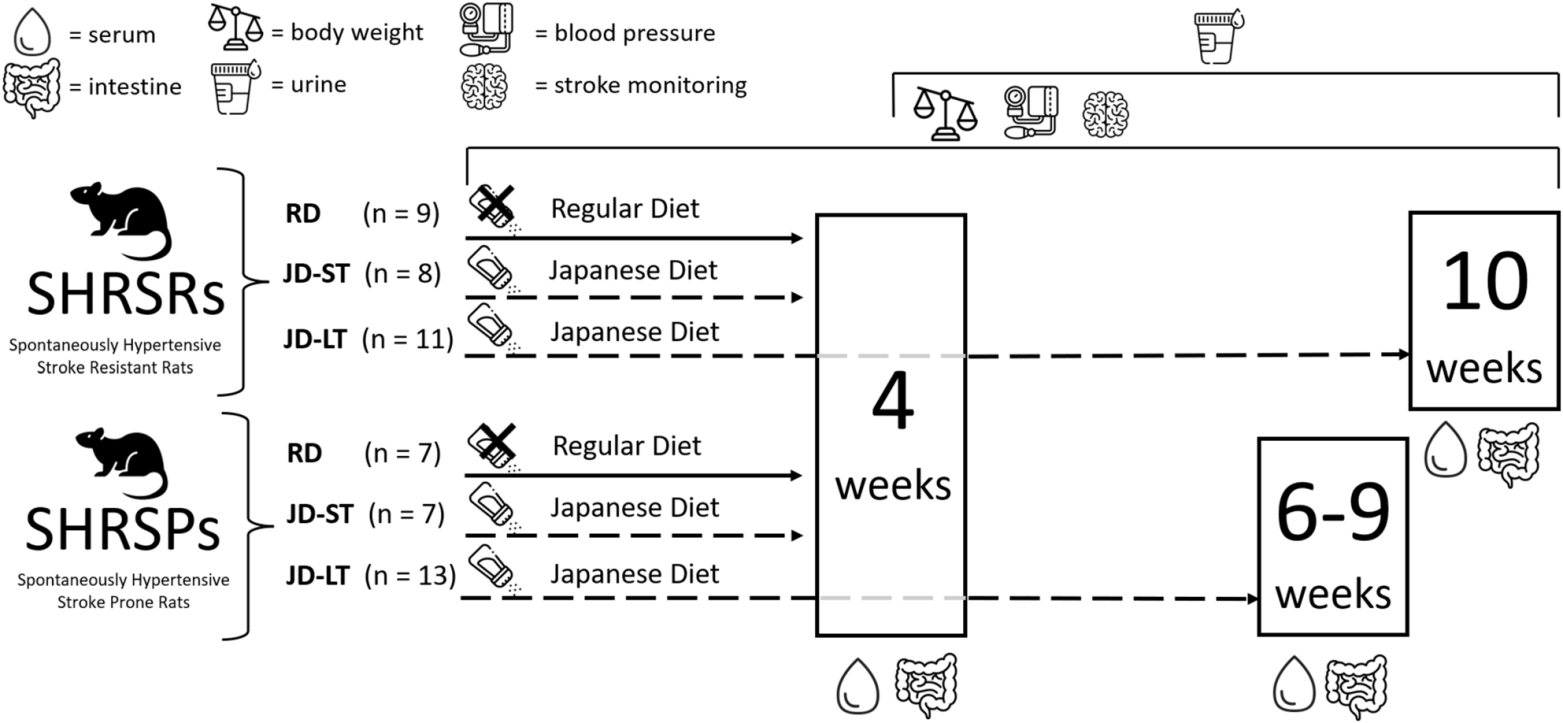
Diagram of experimental groups and samplings. SHRSR and SHRSP animals were sacrificed after 4 weeks of regular diet (RD) (SHRSR RD and SHRSP RD groups, respectively), or after 4 weeks (short term, ST) of Japanese Diet (JD) (SHRSR JD-ST and SHRSP JD-ST groups, respectively). JD-fed rats continued JD (long-term, LT) for 10 weeks before being sacrificed (SHRSR JD-LT group) or until death by stroke, which occurred between week 6 and week 9 (SHRSP JD-LT group). Serum, large and small intestines, and kidneys were collected after sacrifice. Body weight of each animal was measured weekly from the beginning of the diet until sacrifice, and blood pressure and proteinuria were measured during the 4th, 6th, 8th and 10th week of the diet. Stroke onset was continuously monitored throughout the experiment. The figure was created using icons from https://www.freepik.com/.

Male SHRSPs (n = 27) and SHRSRs (n = 28) were housed in the animal facility at the Neuromed Institution (Pozzilli, Italy). Rats were kept 2 or 3 per cage under controlled conditions of light, temperature, and humidity. For the first six weeks of life, the rats received a standard rat chow diet and had free access to water. Then, rats of both strains were randomly divided into two subgroups: the first subgroup (SHRSR, n = 9 and SHRSP, n = 7) received a control diet (regular diet – RD, containing 22% protein, 2.7 mg/g Na + , 7.4 mg/g K + , 0.05 mg/g methionine), and the second subgroup (SHRSR, n = 19 and SHRSP, n = 20) received a high-salt/low-potassium Japanese style diet – JD, containing 17.5% protein, 3.7 mg/g Na + , 6.3 mg/g K + , 0.03 mg/g methionine, with the addition of 1% NaCl in the drinking water. Both RD and JD were provided by Lab. Piccioni, Milan, Italy. After 4 weeks of either RD or JD (short-term, ST), rats of both strains were sacrificed (SHRSP RD, n = 7; SHRSP JD-ST, n = 7; SHRSR RD, n = 9; SHRSR JD-ST, n = 8). The remaining rats continued JD (long-term, LT) until week 10 (SHRSR JD-LT, n = 11) or until death by stroke, which occurred between week 6 and week 9 (SHRSP JD-LT, n = 13) (Fig. 8). Rats were regularly monitored throughout the experiment for BW, systolic BP levels (measured by tail-cuff sphygmomanometry), proteinuria levels, diarrhoea occurrence, and signs of stroke (paresis/hemiparesis of limbs, epileptic attacks, sudden death). At the end of the appointed dietary regimen or at the time of stroke, the animals were sacrificed, urine and serum samples were collected, and the large and small intestines were removed, washed in PBS, and stored at -80°C until analysis. A portion of the large intestine was cut longitudinally, and the content was collected and stored at – 80 °C for microbiota profiling. A portion of the small intestine was cut longitudinally, the content was removed, the lumen was washed with PBS and the mucosa was collected by scraping the intestinal walls before storage at – 80 °C until mRNA analysis. Rats were sacrificed by decapitation under anesthesia (isoflurane, 5% for induction, and 2% for maintenance). Histological examination of kidneys of SHRSRs and SHRSPs was performed as previously reported by Rubattu et al.^38^. All animal studies were performed in accordance with approved protocols by the IRCCS Neuromed Animal Care Review Board and the “Istituto Superiore di Sanità” (ISS permit number: 1086/2020) and were conducted according to EU Directive 2010/63/EU for animal experiments. The study is reported in accordance with the ARRIVE guidelines (https://arriveguidelines.org).

### Gut microbiota analysis: microbial DNA extraction and 16S rRNA amplicon sequencing

Microbial DNA was extracted from about 200 mg of large intestine content using the QIAamp DNA Stool Mini Kit (Qiagen, Hilden, Germany) with minor modifications as described in Costabile et al.^39^. Briefly, samples were suspended in 1.4 ml of ASL buffer and incubated at 95 °C for 5 min. Samples were then treated two times in a FastPrep-24 instrument (MP Biomedicals, Irvine, CA, USA) at 5.5 movements per sec for 45 s, after the addition of four 3-mm glass beads and 0.5 g of 0.1-mm zirconia beads (BioSpec Products, Bartlesville, OK, USA), and kept on ice for 5 min between treatments. After centrifugation at 15,000×*g* for 1 min at room temperature, half of the InhibitEX tablet was added to 1.2 ml of the supernatant and samples were mixed vigorously before incubation at room temperature for 2 min. DNA extraction was then continued following the manufacturer’s instructions (Qiagen). DNA concentration and quality were assessed using a NanoDrop ND-1000 spectrophotometer (NanoDrop Technologies, Wilmington, DE, USA).

Libraries of the V3–V4 hypervariable regions of the 16S rRNA gene were prepared using the 341F and 785R primers with Illumina adapter overhang sequences^40^ and following the “16S Metagenomic Sequencing Library Preparation” protocol (Illumina, San Diego, CA, USA). The final libraries, indexed and purified, were pooled at an equimolar concentration, denatured, and diluted to 5 pM before sequencing on an Illumina MiSeq platform with a 2 × 250 bp paired-end protocol according to the manufacturer’s instructions (Illumina).

### Serum calprotectin levels

To evaluate the inflammatory status of the gut, serum calprotectin levels were assessed using the Rat Calprotectin (CALP) ELISA Kit (Cusabio, Houston, TX, USA) according to the manufacturer’s protocols. The reported kit sensitivity is 15.6 ng/ml.

### RNA isolation and reverse transcription-quantitative PCR (RT-qPCR) for ZO-1 and Ocln

To investigate the integrity of the gut epithelial barrier, ZO-1 and Ocln gene expression levels were measured in the small intestine. Total RNA was extracted from 50 mg of small intestinal mucosa. The tissue was added with 1 ml of TRIzol™ Reagent (Life Technologies, Carlsbad, CA, USA) and three 3-mm glass beads (BioSpec Products), and homogenized twice in a tissue lyser (Tissue Lyser LT, Qiagen) at 50 Hz for 30 s. After the addition of 200 µl of chloroform, the suspension was mixed well and incubated at room temperature for 10 min. The samples were centrifuged at 12,000×*g* for 15 min at 4 °C, and 400 µl of the aqueous phase was collected and mixed with 350 µl of 70% ethanol. The resulting solution was applied to the NucleoSpin® RNA Column from the NucleoSpin RNA kit (Macherey–Nagel GmbH & Co. KG, Düren, Germany) and RNA was purified according to the manufacturer’s instructions. RNA was spectrophotometrically quantified (260 nm) (DeNovix Inc., Wilmington, DE, USA) and its quality determined by gel electrophoresis on 1% agarose. Then, 1 µg of RNA was reverse-transcribed to cDNA using 5X iScript RT Supermix (Bio-Rad Laboratories Inc., Hercules, CA, USA) in a final volume of 20 μl. qPCR was carried out using a CFX 96 Real Time System (Bio-Rad Laboratories Inc.) and iTaq Universal SYBR® Green Supermix (Bio-RAD Laboratories Inc.). All samples were analyzed in duplicate, and RT-qPCR assays were carried out for three different reference genes, *Actb* (Actin beta), *GAPDH* (Glyceraldehyde-3-phosphate dehydrogenase) and *Pgk1* (Phosphoglycerate kinase 1), and the target genes *ZO-1* and *Ocln*. Primers were either designed using Beacon Designer 2.07 (Premier Biosoft International, Palo Alto, CA, USA) or taken from the literature (Supplementary Table S2). The specificity of the amplified PCR products was verified by melting curve analysis and agarose gel electrophoresis. *ZO-1* and *Ocln* mRNA data were normalized based on the geometric mean of the Ct of the three reference genes (ΔCt = Ct _mean ref. genes_–Ct _interest gene_). For each strain, the relative gene expression of the studied genes in JD-fed animals was calculated as fold change using the 2^−ΔΔCt^ method in relation to the RD T0 time point (ΔΔCt = ΔCt_T0 JD_ or _T1 JD_–ΔCt_T0 RD_)^41^. Undetectable RT-qPCR Cts were assigned a value of 40 to avoid overestimation of means.

### Bioinformatics and statistical analysis

For microbiota analysis, raw sequences were processed using a pipeline combining PANDAseq^42^ and QIIME 2^43^. Specifically, paired-end sequences were first assembled into a single-end amplicon using PANDAseq, retaining only assembled reads in the range of 350 to 550 nucleotides. An error correction step was then performed using the USEARCH11 ‘fastq filter’ module^44^ with a maximum error rate of 0.03 to discard low-quality sequences. Sequences were then binned into amplicon sequence variants (ASVs) using the DADA2 pipeline^45^ with chimeras removed at the same time. Taxonomic assignment was performed using the VSEARCH algorithm^46^ to align ASVs to the Greengenes database version 13.8. Alpha diversity was calculated using several metrics, including the Shannon index and the number of observed ASVs. Beta diversity was estimated based on unweighted and weighted UniFrac distances and visualized on Principal Coordinates Analysis (PCoA) plots.

All statistical analyses were performed using R software (version 4.2.2; R Core Team 2022) and JASP (JASP Team 2022, version 0.16.2.0).

Animal BW was analyzed by two-way ANOVA followed by Tukey post-hoc comparison. Differences in proteinuria and systolic BP levels were tested by two-way ANOVA or one-way ANOVA. Tubulo-interstitial fibrosis inter-group means were compared using Mann–Whitney test with Bonferroni’s correction. Survivor function in rats monitored over JD feeding was estimated by the life-table method. Log-rank and Mann–Whitney statistics were used for testing equality of survivor function. Calprotectin and gene expression data were analyzed by *t*-test or two-way ANOVA, followed by Tukey post-hoc comparison, to investigate the influence of the strain and diet.

For the gut microbiota, PCoA plots were generated using the R packages “vegan” (http://www.cran.r-project.org/package=vegan/) and “Made4”^47^ and data separation was tested by a permutation test with pseudo-F ratios (function “Adonis” in “vegan”). Differences in alpha diversity and relative taxon abundances (at all taxonomic levels) between groups were assessed by Kruskal–Wallis test, followed by post-hoc Mann–Whitney *U* test. Kendall rank correlation test was used to assess associations between relative taxon abundances and BP and proteinuria levels. Only statistically significant correlations with absolute Kendall’s tau ≥ 0.2 were considered.

### Supplementary Information

Supplementary Information.

## Data Availability

Sequencing reads were deposited in the National Center for Biotechnology Information Sequence Read Archive (NCBI SRA; BioProject ID PRIJNA1101725).
