# Supplementary material for: Early and late gut microbiota signatures of stroke in high salt-fed stroke-prone spontaneously hypertensive rats
Source: Sci Rep. 2024 Aug 23;14:19575. doi: 10.1038/s41598-024-69961-9 (PMC11343747; doi:10.1038/s41598-024-69961-9)
Supplement: Supplementary file 1 — Supplementary Information. [file 41598_2024_69961_MOESM1_ESM.docx]

**Early and late gut microbiota signatures of stroke in high salt-fed stroke-prone spontaneously hypertensive rats**

Silvia Bencivenni^1,2^, Sara Roggiani^1,3^, Augusta Zannoni^2,4^, Gabriele Conti^1,3^, Marco Fabbrini^3^, Maria Cotugno^5^, Rosita Stanzione^5^, Donatella Pietrangelo^6^, Margherita Litterio^5^, Maurizio Forte^5^, Carla Letizia Busceti^5^, Francesco Fornai^5,7^, Massimo Volpe^6,8^, Silvia Turroni^1^, Patrizia Brigidi^3,4^, Monica Forni^3,4^, Speranza Rubattu^5,6,#^, Federica D’Amico^1,*,#^

(1) Unit of Microbiome Science and Biotechnology, Department of Pharmacy and Biotechnology, University of Bologna, Bologna

(2) Department of Veterinary Medical Sciences, University of Bologna, Ozzano dell’Emilia (BO)

(3) Department of Medical and Surgical Sciences, University of Bologna, Bologna

(4) Health Sciences and Technologies-Interdepartmental Center for Industrial Research (CIRI-SDV), Alma Mater Studiorum-University of Bologna, 40126 Bologna

(5) IRCCS Neuromed, Pozzilli (Isernia)

(6) Department of Clinical and Molecular Medicine, School of Medicine and Psychology, Sapienza University of Rome, Rome

(7) Department of Translational Research and New Technologies in Medicine and Surgery, University of Pisa, Pisa, Italy

(8) IRCCS San Raffaele, Rome; all from Italy

*Address for correspondence: Federica D’Amico, Unit of Microbiome Science and Biotechnology, Dept. Pharmacy and Biotechnology, University of Bologna, Via Belmeloro 6, 40126 Bologna, Italy. Tel.: +39 051 2099727; email: [federica.damico8@unibo.it](mailto:federica.damico8@unibo.it)

#These authors are joint senior authors

**Supplementary Information**


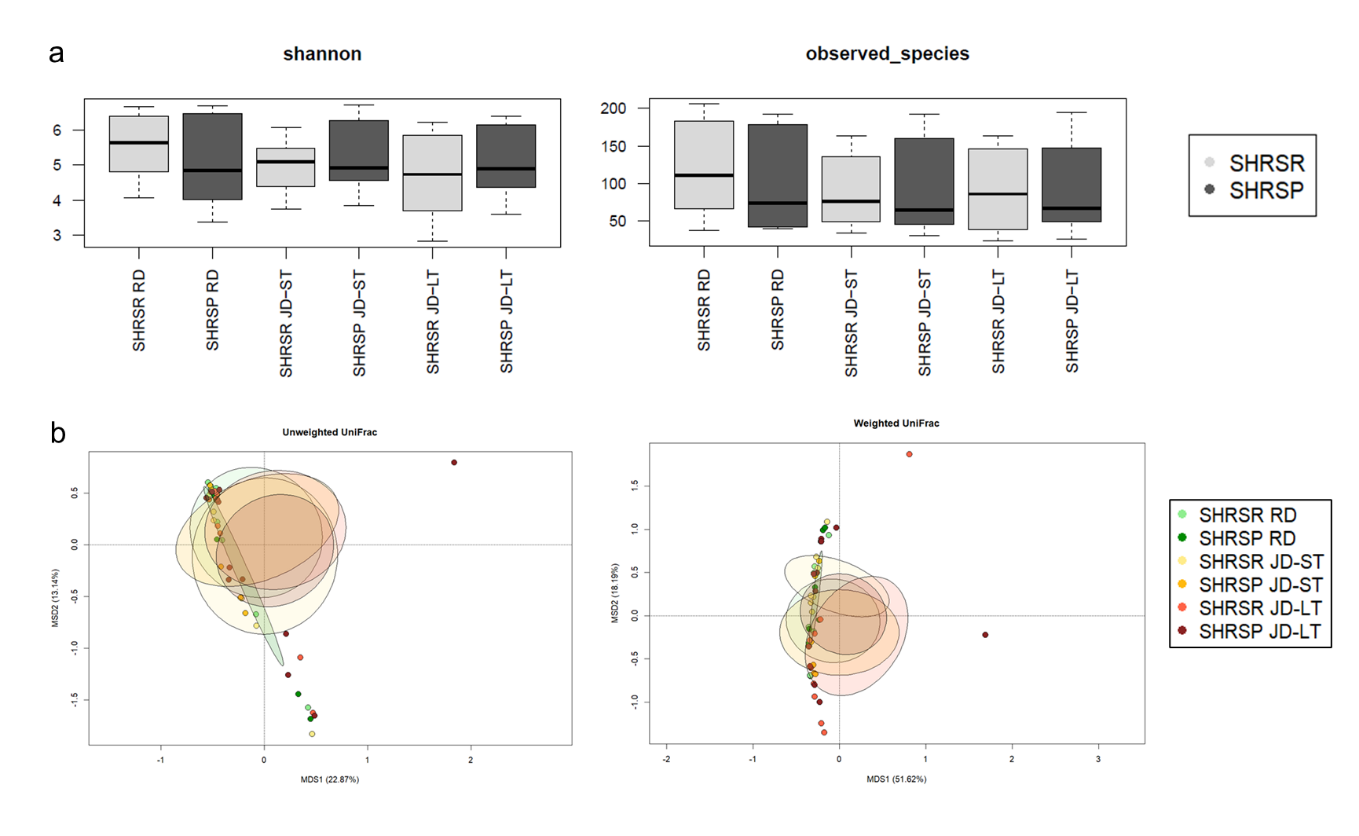


**Figure S1. Gut microbiota diversity of SHRSRs and SHRSPs after 4 weeks of regular diet (RD) or Japanese diet (JD-ST), and after long-term JD feeding (JD-LT).** (a) Boxplots showing the distribution of alpha diversity, calculated according to the Shannon index and the number of observed ASVs, in the two rat strains after 4 weeks of RD or JD feeding (JD-ST) and after long-term JD feeding (JD-LT). No differences were found between the study groups (p≥0.6, Kruskal-Wallis test). (b) Principal Coordinates Analysis based on weighted and unweighted UniFrac distances between the study groups. The ellipses represent the 95% confidence interval for each study group. No segregation between groups was found (p>0.5, adonis). SHRSR RD, n=9; SHRSP RD, n=7; SHRSR JD-ST, n=8; SHRSP JD-ST, n=7; SHRSR JD-LT, n=11; SHRSP JD-LT, n=13.


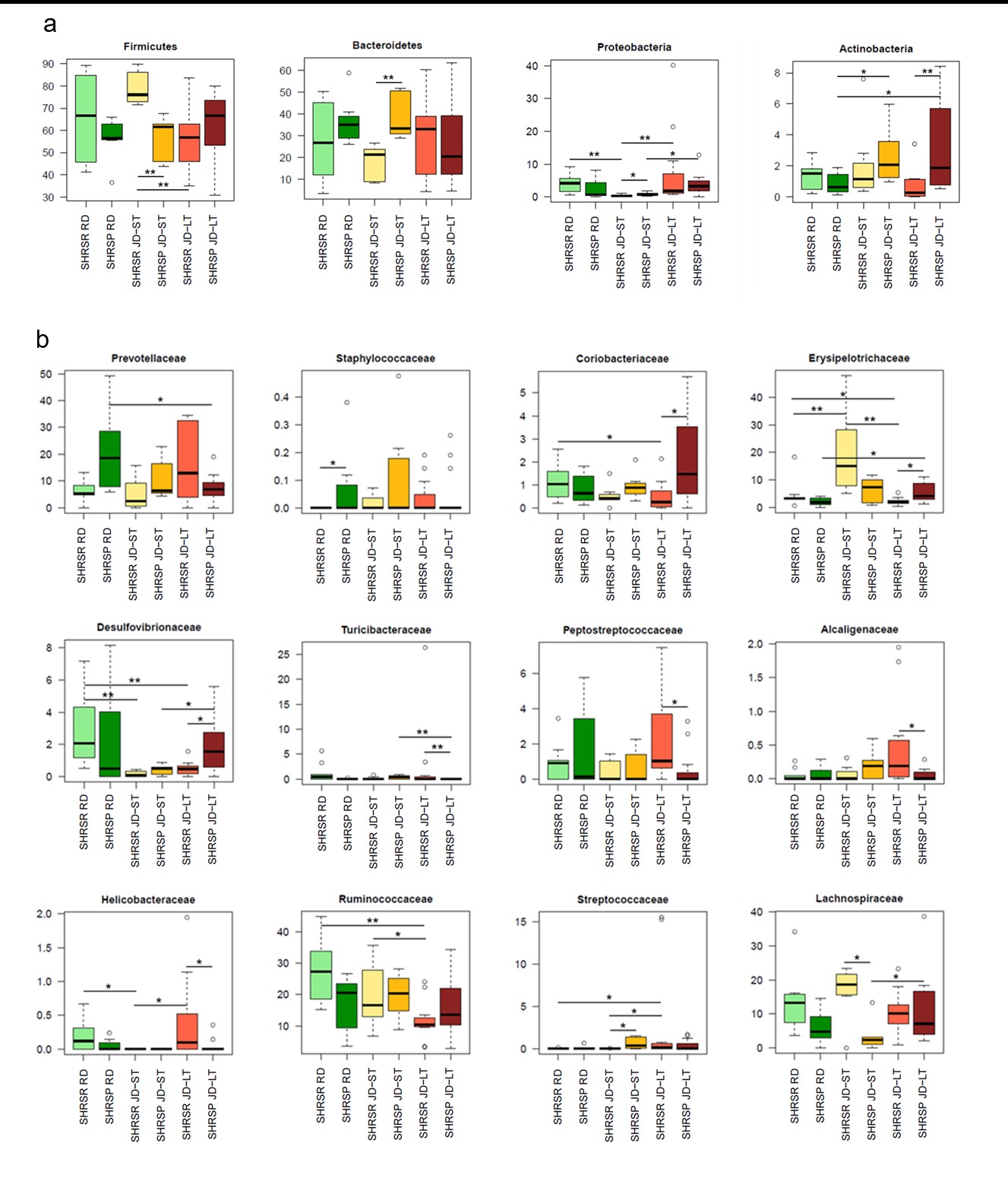


**Figure S2. Gut microbiota signatures of SHRSRs and SHRSPs after 4 weeks of regular diet (RD) or Japanese diet (JD-ST), and after long-term JD feeding (JD-LT).** Boxplots showing the relative abundance distribution of phyla (a) and families (b) differentially represented between the study groups [the two rat strains after 4 weeks of RD or JD feeding (JD-ST) and after long-term JD feeding (JD-LT)]. Mann-Whitney U test; * p<0.05, ** p<0.01. SHRSR RD, n=9; SHRSP RD, n=7; SHRSR JD-ST, n=8; SHRSP JD-ST, n=7; SHRSR JD-LT, n=11; SHRSP JD-LT, n=13.


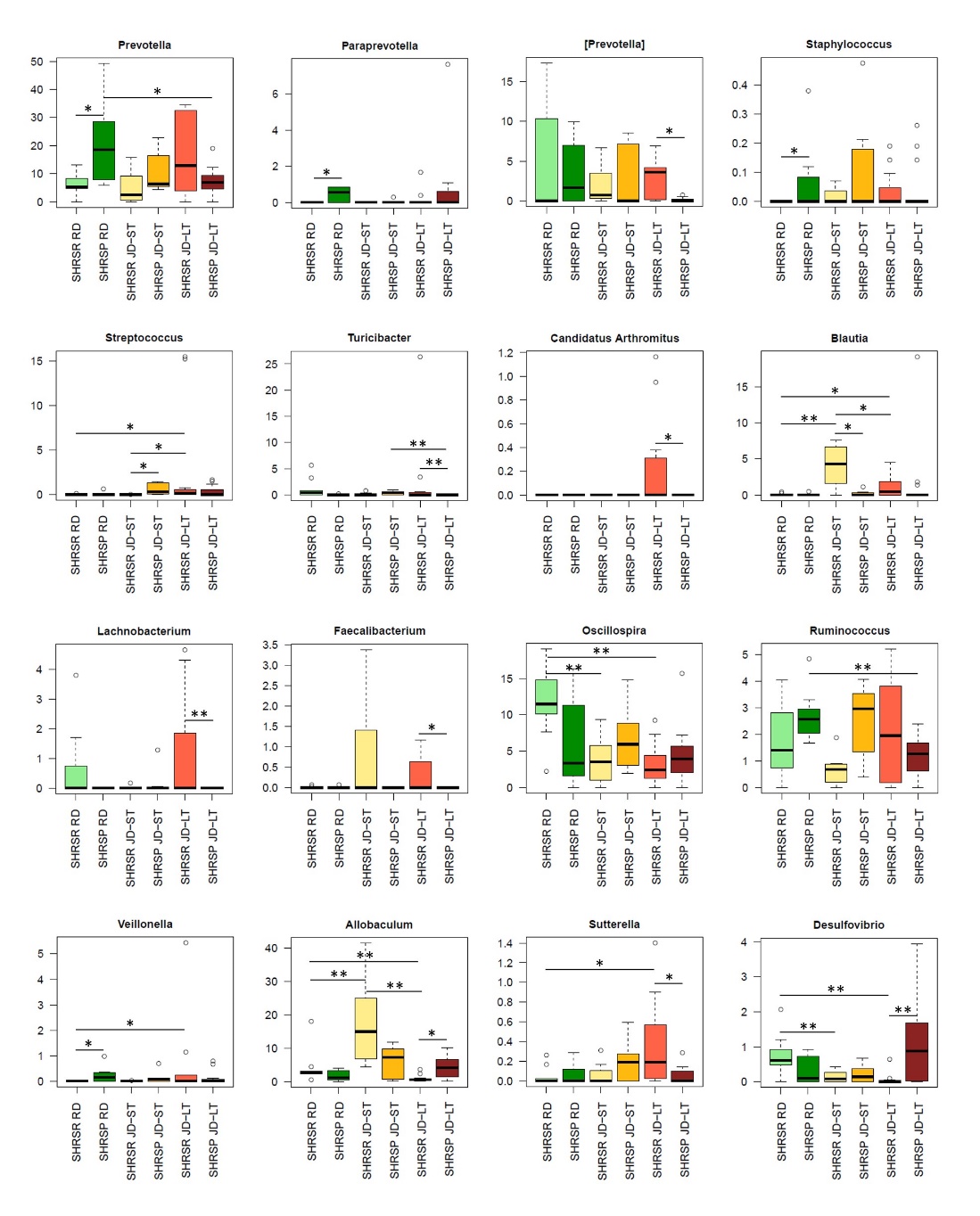


**Figure S3. Genera in the gut microbiota of SHRSRs and SHRSPs that show changes in relative abundance after 4 weeks of regular diet (RD) or Japanese diet (JD-ST), and after long-term JD feeding (JD-LT).** Boxplots showing the relative abundance distribution of genera differentially represented between the study groups [the two rat strains after 4 weeks of RD or JD feeding (JD-ST) and after long-term JD feeding (JD-LT)]. Mann-Whitney U test; * p<0.05, ** p<0.01. SHRSR RD, n=9; SHRSP RD, n=7; SHRSR JD-ST, n=8; SHRSP JD-ST, n=7; SHRSR JD-LT, n=11; SHRSP JD-LT, n=13.

**Table S1.** **Body weight (g) of animals at study onset.**

| **Animal ID** | **Strain** | **Diet** | **Group** | **BW (g)** |
| --- | --- | --- | --- | --- |
| 837 | SHRSR | RD |  | 126 |
| 838 | SHRSR | RD |  | 128 |
| 839 | SHRSR | RD |  | 110 |
| 840 | SHRSR | RD |  | 109 |
| 841 | SHRSR | RD |  | 122 |
| 4745 | SHRSR | RD |  | 121 |
| 4746 | SHRSR | RD |  | 118 |
| 4747 | SHRSR | RD |  | 115 |
| 4748 | SHRSR | RD |  | 117 |
| 1101 | SHRSP | RD |  | 114 |
| 1102 | SHRSP | RD |  | 112 |
| 1103 | SHRSP | RD |  | 115 |
| 1104 | SHRSP | RD |  | 110 |
| 4742 | SHRSP | RD |  | 110 |
| 4743 | SHRSP | RD |  | 112 |
| 4744 | SHRSP | RD |  | 108 |
| 1105 | SHRSP | JD | ST | 116 |
| 1106 | SHRSP | JD | ST | 109 |
| 1107 | SHRSP | JD | ST | 117 |
| 1108 | SHRSP | JD | ST | 113 |
| 5848 | SHRSP | JD | ST | 112 |
| 5850 | SHRSP | JD | ST | 110 |
| 5851 | SHRSP | JD | ST | 109 |
| 1397 | SHRSR | JD | ST | 110 |
| 1398 | SHRSR | JD | ST | 128 |
| 1399 | SHRSR | JD | ST | 115 |
| 1400 | SHRSR | JD | ST | 117 |
| 5855 | SHRSR | JD | ST | 120 |
| 5856 | SHRSR | JD | ST | 119 |
| 5857 | SHRSR | JD | ST | 121 |
| 2921 | SHRSP | JD | LT | 111 |
| 2922 | SHRSP | JD | LT | 113 |
| 2923 | SHRSP | JD | LT | 113 |
| 2924 | SHRSP | JD | LT | 94 |
| 2925 | SHRSP | JD | LT | 94 |
| 2926 | SHRSP | JD | LT | 106 |
| 2927 | SHRSP | JD | LT | 104 |
| 2928 | SHRSP | JD | LT | 102 |
| 5852 | SHRSP | JD | LT | 114 |
| 5853 | SHRSP | JD | LT | 115 |
| 5791 | SHRSP | JD | LT | 112 |
| 5792 | SHRSP | JD | LT | 110 |
| 5794 | SHRSP | JD | LT | 112 |
| 5858 | SHRSR | JD | LT | 119 |
| 5859 | SHRSR | JD | LT | 117 |
| 5860 | SHRSR | JD | LT | 115 |
| 5787 | SHRSR | JD | LT | 115 |
| 5788 | SHRSR | JD | LT | 117 |
| 5789 | SHRSR | JD | LT | 117 |
| 5790 | SHRSR | JD | LT | 118 |
| 2933 | SHRSR | JD | LT | 115 |
| 2934 | SHRSR | JD | LT | 117 |
| 2935 | SHRSR | JD | LT | 118 |
| 2936 | SHRSR | JD | LT | 110 |

*BW, Body weight; JD, Japanese diet; LT, long-term; RD, regular diet; SHRSP, Spontaneously Hypertensive Stroke Prone rats; SHRSR, Spontaneously Hypertensive Stroke-Resistant; ST, short-term*

**Table S2.** **List of primer pairs used for RT-qPCR, along with amplicon size (bp) and accession number in the NCBI (National Center of Biotechnology Information) database.**

| Gene |  | Primer sequence (5'>3') | Amplicon size (bp) | Accession number | Reference |
| --- | --- | --- | --- | --- | --- |
| *ZO-1* | F CACGATGCTCAGAGACGAAGG | | 231 | NM _001106266.1 | *Wu et al., 2020*^17^ |
|  | R CTGTATGGTGGCTGCTCAAGG | |  |  |  |
| *Ocln* | F | TCTTTGTATAAGTCACCGCCTCTG | 185 | NM_031329 | *Present study* |
|  | R | GTTTCATAGTGGTCTGGGTCTGTC |  |  |  |
| *Actb* | F | GGTCAGGTCATCACTATCGGCAATG | 165 | NM_031144.3 | *Present study* |
|  | R | CAGCACTGTGTTGGCATAGAGGTC |  |  |  |
| *GAPDH* | F | GGCAAGTTCAACGGCACAGTCAAG | 128 | NM_017008.4 | *Present study* |
|  | R | ACGACATACTCAGCACCAGCATCAC |  |  |  |
| *Pgk1* | F | GGGTATTTGAATGGGAAGCCTTTGC | 106 | NM_053291.3 | *Present study* |
|  | R | GTGTCTCCGCCTCCTATGATAGTG |  |  |  |

*ZO-1, Zonulin-1; Ocln, Occludin; Actb, Actin beta; GAPDH, Glyceraldehyde-3-phosphate dehydrogenase; Pgk1, Phosphoglycerate kinase 1.*
